# Phytochemicals and Enzyme Inhibitory Capacities of the Methanolic Extracts from the Italian Apple Cultivar Mela Rosa dei Monti Sibillini

**DOI:** 10.3390/ph13060127

**Published:** 2020-06-22

**Authors:** Víctor López, Francisco Les, Serena Mevi, Joice Guileine Nkuimi Wandjou, Guillermo Cásedas, Giovanni Caprioli, Filippo Maggi

**Affiliations:** 1 Department of Pharmacy, Faculty of Health Sciences, Universidad San Jorge, Villanueva de Gállego, 50830 Zaragoza, Spain; fles@usj.es (F.L.); gcasedas@usj.es (G.C.); 2 Instituto Agroalimentario de Aragón-IA2 (CITA-Universidad de Zaragoza), 50013 Zaragoza, Spain; 3 School of Pharmacy, University of Camerino, 62032 Camerino, Italy; serena.mevi@studenti.unicam.it (S.M.); joice.nkuimiwandjou@unicam.it (J.G.N.W.); giovanni.caprioli@unicam.it (G.C.)

**Keywords:** polyphenols, triterpenes, antioxidants, enzyme inhibitors, apple, functional foods

## Abstract

The phytochemical profile of the methanolic extracts (pulp and peel) obtained from two dehydration methods (drying and freeze-lyophilization) of the traditional Italian apple Mela Rosa dei Monti Sibillini, as well as their inhibitory properties against some biological enzymes (α-glucosidase, lipase, monoamine oxidase A, tyrosinase and acetylcholinesterase) were assessed in this study. HPLC-DAD-MS technique was used for the determination of polyphenolic and triterpenic compounds. The determination of the enzymes inhibitory effect was made through spectrophotometric techniques. The peel extracts were richer in bioactive compounds than the pulp. In this regard, the extracts from freeze-lyophilization displayed higher levels of flavan-3-ols, flavonol glycosides and dihydrochalcones. However, the extracts obtained from dried material displayed a stronger enzyme inhibition. Notably, the peel extracts showed a higher activity than the pulp ones, especially in terms of α-glucosidase whereby some samples exerted a similar enzymatic inhibition than acarbose (100% inhibition) at high concentrations (1 mg/mL). These results encourage thus further studies on this traditional Italian apple as a potential source of nutraceuticals helpful to prevent the insurgence of some pathologies.

## 1. Introduction

The high consumption of fruits and vegetables has been associated to a good health state. Indeed, it is reported that the low mortality rate due to fruit and vegetable consumption is due to the phytochemicals they contain [1]. Apple fruit (*Malus domenica* Borkh) have shown to be one of the most important dietary sources of polyphenols, whose consumption has been associated with human wellness [2]. It has also been reported that traditional and overlooked apples were proved to be higher sources of phytonutrients than the commercial ones [3]. Despite their particularity (shape, taste, nutritional values), these old apples are on the verge of extinction because of globalization. Hence, the characterization of old apple cultivars is important to enhance their value and increase their production [4,5].

Polyphenols and triterpenes are secondary metabolites usually found in the apple varieties. Five classes of polyphenols are reported for this fruit: flavan-3-ols/proanthocyanidins (catechin, epicatechin, procyanidin of the group A and B), flavonols (quercetin, rutin, kaempferol), dihydrochalcones (phloretin and phloridzin), hydroxycinnamic acids (chlorogenic acid, *p*-coumaric acid, caffeic acid, ferulic acid) and hydroxybenzoic acids (gallic acid) [6]. The triterpene acids, generally found in the peel, are mainly represented by ursolic acid, oleanolic acid, betulinic acid, and annurcoic acid [7,8].

Many pharmaceuticals of current use with therapeutic applications act mechanistically as enzyme inhibitors. The consumption of dietary polyphenols is linked to the reduction of the incidence of neurodegenerative diseases such as Alzheimer’s disease (AD) [9]. Some plant extracts rich in polyphenols as those from green tea and blueberry have demonstrated some inhibitory activities against acetylcholinesterase (AChE) exhibiting thus neuroprotective capacity [9]. α-Glucosidase inhibitors are currently used in the management of type 2 diabetes and hyperglycemia [10]. Polyphenols from plant extracts have shown to be efficient α-glucosidase and amylase inhibitors and also to block glucose absorption by the inhibition of the Na^+^- dependent glucose transporters, SGLT1 and SGLT2; contributing thus to the prevention of hyperglycemia [10]. One of the strategies of the prevention of obesity, which is becoming a global health issue, is the reduction of the activity of pancreatic lipase, which is involved in the digestion of triglycerides into monoglycerides and free fatty acids readily absorbable [11]. It is reported that many extracts from foods potentially inhibit lipase enzyme [11]. Many plant polyphenols have also shown inhibitory properties against monoamine oxidase A (MAO-A), an enzyme involved in depression [12,13]. It is suggested that the skin-lightening effect of the crude polyphenolic extract of the acerola fruit and other natural products may be due the tyrosinase activity inhibition in melanocytes, suppressing thus melanogenesis [14,15]. Other enzymes such as 5α-reductase and carbonic anhydrase can also be inhibited by some polyphenols [16,17]. The study of the inhibitory effect of polyphenols on the biological enzymes can be an important way to counteract and manage many diseases.

The Mela Rosa dei Monti Sibillini (MR) is an ancient apple cultivated since the age of the Roman empire in central Italy at an altitude between 400 and 900 m [4,5]. Due to its uniqueness given by its particular shape, smell, taste and shelf life, this apple is regaining attention. To recover and preserve the germplasm of this overlooked old variety, many actions such as the increase of its cultivation and research of the health benefits have been put in place by the local authorities. Our previous studies also showed that samples of this cultivar have a high content of polyphenols and triterpenes such as annurcoic acid which was firstly found in the Annurca cultivar [4,7]. 

The consumption of the hydroalcoholic extracts of this cultivar has shown protective effects against renal ischemia/reperfusion injury and CCl_4_-induced hepatotoxicity in rats [18,19].

The development of nutraceuticals from fruits and vegetables to be employed in the prevention and treatment of different diseases is a great challenge and can lead to huge benefits for the health and the environment [11]. On this basis, the aim of this work was to assess the inhibitory effects of the peel and pulp methanolic extracts of the Mela Rosa dei Monti Sibillini obtained from dried (DEPe and DEPu respectively) and lyophilised (LEPe and LEPu, respectively) material on different enzymes, namely α-glucosidase, lipase, MAO-A, AChE and tyrosinase (TYR).

## 2. Results

### 2.1. Polyphenols and Triterpenes Composition

The HPLC-DAD-MS analysis of polyphenolic and triterpenic compounds was carried out on the extracts obtained through two different dehydration methods, i.e., drying at 45 ± 5 °C and freeze-lyophilization. The monitored compounds were flavan-3-ols (catechin, epicatechin, procyanidin A2 and procyanidin B2), flavonols (rutin, quercetin, quercetin-3-D-galactoside, kaempferol and kaempferol-3-glucoside), anthocyanins (cyanidin-3-glucoside), hydroxycinnamic acids (*p*-coumaric acid, neochlorogenic acid, chlorogenic acid, caffeic acid and *trans*-ferulic acid), hydroxybenzoic acid (gallic acid), dihydrocalchones (phloretin and phloridzin) and triterpenes (oleanolic and ursolic acids) as reported in Table 1 and Table 2.

HPLC analysis showed that the LEPe (8160.7–12687.5 mg/kg) were richer in polyphenols than DEPe (4520.3–6868.8 mg/kg). Whereas, triterpenes seem to be higher in DEPe (1960.2–21390.4 mg/kg) than in LEPe (903.9–18736.0 mg/kg). The most represented class was triterpenes, followed by flavan-3-ols and dihydrochalcones. In the samples, the most abundant compounds were epicatechin (1740.2–3999.0 and 938.1–2083.5 mg/kg for LEPe and DEPe, respectively), procyanidin B2 (1655.9–2732.7 and 855.0–2041.4 mg/kg for LEPe and DEPe, respectively) and phloridzin (730.2–1809.5 and 387.7–1315.0 mg/kg for LEPe and DEPe, respectively) with an exception for some samples which exhibited a high content of chlorogenic acid (sample 2: 1726.3 and 1551.7 mg/kg for LEPe and DEPe, respectively), quercetin-3-D-galactoside (sample 5: 1313.8 mg/kg for LEPe) and rutin (sample 4:656.7 mg/kg for DEPe; sample 7: 1422.4 and 553.2 mg/kg for LEPe and DEPe, respectively). Ursolic acid was the most abundant phytoconstituent in all samples (583.4–12541.8 mg/kg for LEPe and 1361.7–15088.8 mg/kg for DEPe) apart from sample 2 of LEPe and sample 5 of DEPe where oleanolic acid was present at higher concentrations (10835.5 and 2340.1 mg/kg respectively). The samples with the highest content of the phytochemicals analyzed were samples 6 (31423.5 mg/kg), 2 (26890.9 mg/kg) and 7 (23274.6 mg/kg) for the LEPe, and samples 1 (28250.3 mg/kg), 7 (20256.2 mg/kg) and 2 (20056.9 mg/kg) for DEPe.

In pulp extract analyses, as in the case of peel, the LEPu had a higher content of polyphenol compounds than the DEPu. The most abundant classes present in both DEPu and LEPu were flavan-3-ols and hydroxycinnamic acids. Epicatechin (494.5–1725.2 mg/kg for LEPu and 586.9–1410.9 mg/kg for DEPu), chlorogenic acid (167.9–1946.8 mg/kg for LEPu and 136.0–1438.7 mg/kg for DEPu) and procyanidin B2 (292.1–1262.2 mg/kg for LEPu and 325.6–1015.6 mg/kg for DEPu) were more concentrated in almost all the samples with an exception of the LEPu samples 5, 7 and 8, and DEPu samples 4 and 5 which also showed a high content of catechin (340.1, 195.1 and 416.9 mg/kg for LEPu; 144.9 and 285.3 mg/kg for DEPu). The samples with the highest content of all bioactive compounds analyzed were samples 8 (4242.2 mg/kg), 2 (3408.4 mg/kg) and 5 (3317.6 mg/kg) for LEPu, and samples 8 (3749.6 mg/kg), 6 (3246.6 mg/kg) and 5 (2902.7 mg/kg) for DEPu.

This analysis also showed variability between the samples studied. In the DEPe, a variation of 15.2% of the total polyphenol was noticed and was similar to that of the LEPe. Whereas in the LEPe, the triterpenes content varied highly in respect to that of the DEPe (83.7 and 56.7% respectively). In the pulp samples, the variability was quite similar for both total polyphenols (29.4 and 31.1% for DEPu and LEPu respectively) and triterpenes concentration (41.2 and 41.8% for DEPu and LEPu respectively).

### 2.2. Enzymatic Inhibitory Effects

#### 2.2.1. α-Glucosidase Inhibition

**Figure 1:**
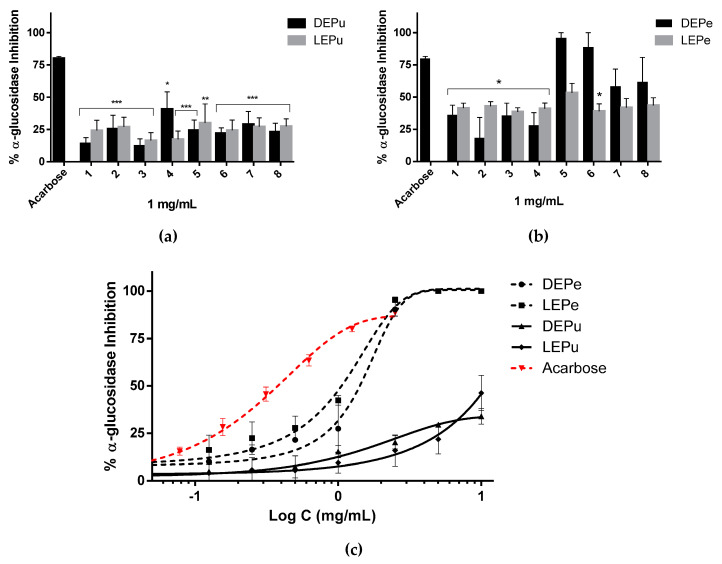
α-Glucosidase inhibition: (**a**) inhibition performed by extracts obtained from pulp (*n* = 6); (**b**) inhibition performed by extracts obtained from peel (*n* = 6); (**c**) inhibition performed by sample 5 for DEPe and DEPu, sample 6 for LEPe and LEPu, and acarbose in a concentration dependent manner (*n* = 3). One-way analysis of variance (ANOVA) followed by Dunnet’s test for multiple comparisons: * *p* < 0.05; ** *p* < 0.01; *** *p* < 0.001 against acarbose. DEPe: dried peel methanolic extract; LEPe: lyophilized peel methanolic extract; DEPu: dried pulp methanolic extract; LEPu: lyophilized pulp methanolic extract.

From the results, both DEPu and LEPu at concentrations of 1 mg/mL inhibited the enzyme at a percentage less than 50% (Figure 1a). Similarly, almost all LEPe (exception with sample 5) and samples 1, 2, 3 and 4 of DEPe have shown an inhibitory effect less than 50%. While at the same concentration (1 mg/mL), DEPe from samples 5 and 6 inhibited α-GLU with a percentage higher than that of acarbose (90% and 80%, respectively) (Figure 1b). DEPe and LEPe inhibited α-GLU in a concentration dependent manner (Figure 1c). IC_50_ values calculated by nonlinear regression are reported in Table 3.

#### 2.2.2. Lipase Inhibition

**Figure 2:**
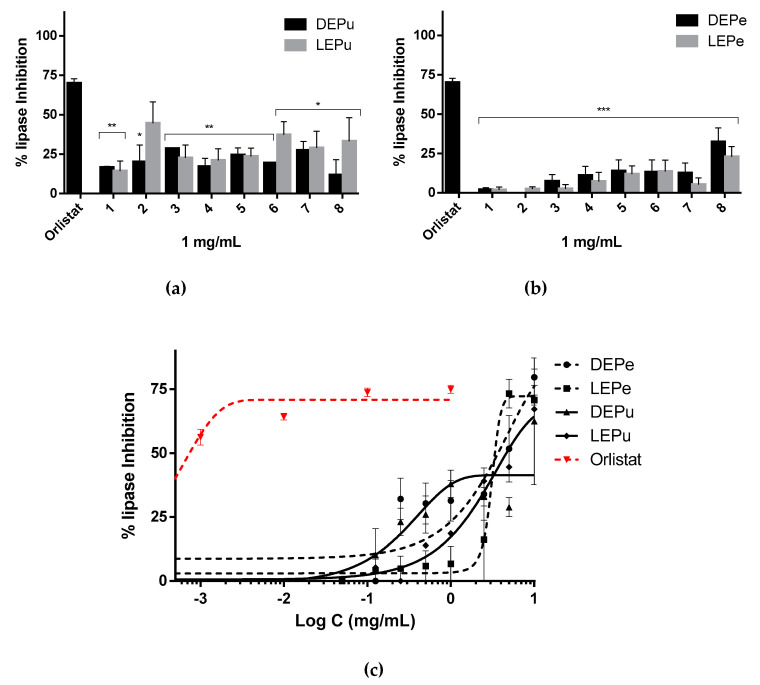
Lipase inhibition: (**a**) inhibition performed by extracts obtained from pulp (*n* = 3); (**b**) inhibition performed by extracts obtained from peel (*n* = 3); (**c**) inhibition performed by by sample 5 for DEPe and DEPu, sample 6 for LEPe and LEPu, and orlistat in a concentration dependent manner (*n* = 3). One-way analysis of variance (ANOVA) followed by Dunnet’s test for multiple comparisons: * *p* < 0.05; ** *p* < 0.01; *** *p* < 0.001 against orlistat. DEPe: dried peel methanolic extract; LEPe: lyophilized peel methanolic extract; DEPu: dried pulp methanolic extract; LEPu: lyophilized pulp methanolic extract.

The extracts showed lipase inhibitory activity (Figure 2c). Indeed, at concentration of 1 mg/mL lipase was inhibited at a percentage lower than 50% by all the samples even if the pulp extracts in this case seemed more active than the peel ones (Figure 2a,b). In contrast, at 10 mg/mL of DEPe, the inhibition obtained was 100%. IC_50_ values were calculated by nonlinear regression (Table 3).

#### 2.2.3. MAO-A Inhibition

**Figure 3:**
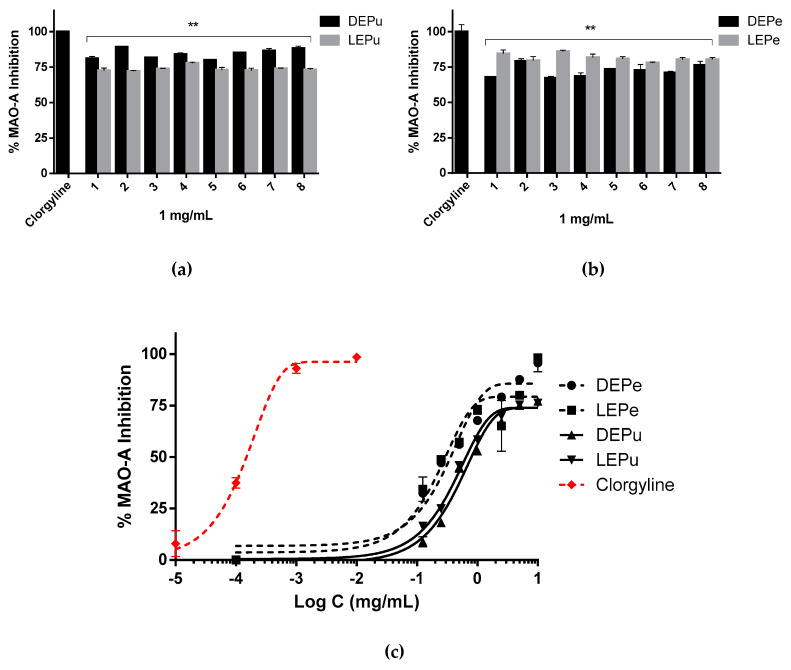
MAO-A inhibition: (**a**) inhibition performed by extracts obtained from pulp (*n* = 3); (**b**) inhibition performed by extracts obtained from peel (*n* = 3); (**c**) inhibition performed by sample 8 obtained from pulp, peel and clorgyline in a concentration dependent manner (*n* = 3). One-way analysis of variance (ANOVA) followed by Dunnet’s test for multiple comparisons: ** *p* < 0.01 against clorgyline. DEPe: dried peel methanolic extract; LEPe: lyophilized peel methanolic extract; DEPu: dried pulp methanolic extract; LEPu: lyophilized pulp methanolic extract.

The extracts were able to inhibit MAO-A in a dose-dependent manner with a similar profile of clorgyline (Figure 3c). The percentages of inhibition were between 67% and 88% with all the samples studied at 1 mg/mL of extract concentration (Figure 3a,b). However, there were significant differences between the IC_50_ of the extracts (DEPe: 533 μg/mL; LEPe: 473 μg/mL; DEPu: 1793 μg/mL and LEPu: 846 μg/mL) and the reference (0.2 μg/mL), calculated by nonlinear regression (Table 3).

#### 2.2.4. AChE Inhibition

**Figure 4:**
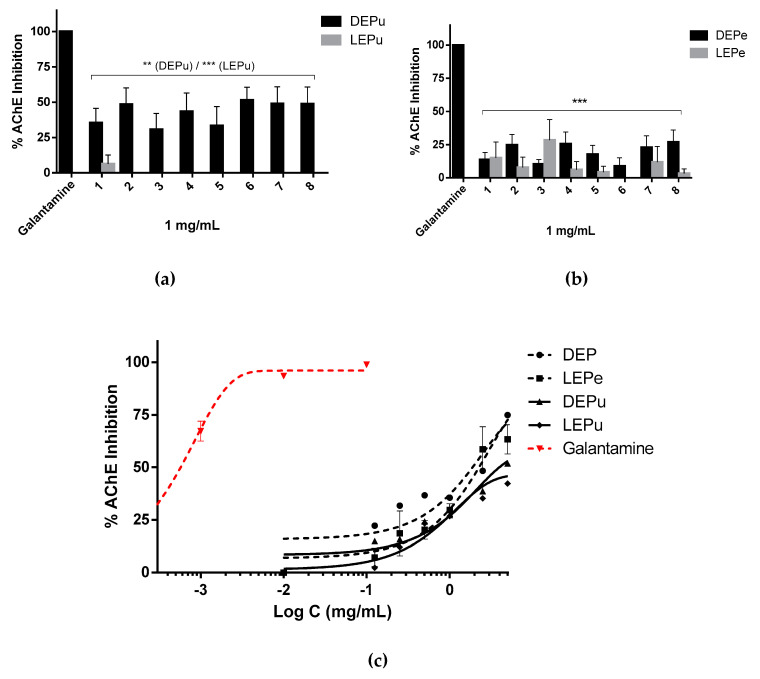
AChE inhibition: (**a**) inhibition performed by extracts obtained from pulp (*n* = 3); (**b**) inhibition performed by extracts obtained from peel (*n* = 3); (**c**) inhibition performed by sample 5 for DEPe and DEPu, sample 6 for LEPe and LEPu, and galantamine in a concentration dependent manner (*n* = 3). One-way analysis of variance (ANOVA) followed by Dunnet’s test for multiple comparisons: ** *p* < 0.01; *** *p* < 0.001 against galantamine. DEPe: dried peel methanolic extract; LEPe: lyophilized peel methanolic extract; DEPu: dried pulp methanolic extract; LEPu: lyophilized pulp methanolic extract.

The extracts had a lower inhibitory effect against AChE than the positive control, galantamine (Figure 4a–c). At 1 mg/mL, the DEPu seems to be more active (inhibition less than 52%) than the DEPe and LEPe (inhibition less than 27%). On the other hand, LEPu showed no activity against the enzyme. Furthermore, the values of IC_50_ obtained from extracts (DEPe: 1889 μg/mL; LEPe: 2261 μg/mL and DEPu: 3963 μg/mL) were significantly different from those achieved by galantamine (Table 3).

#### 2.2.5. TYR Inhibition

**Figure 5:**
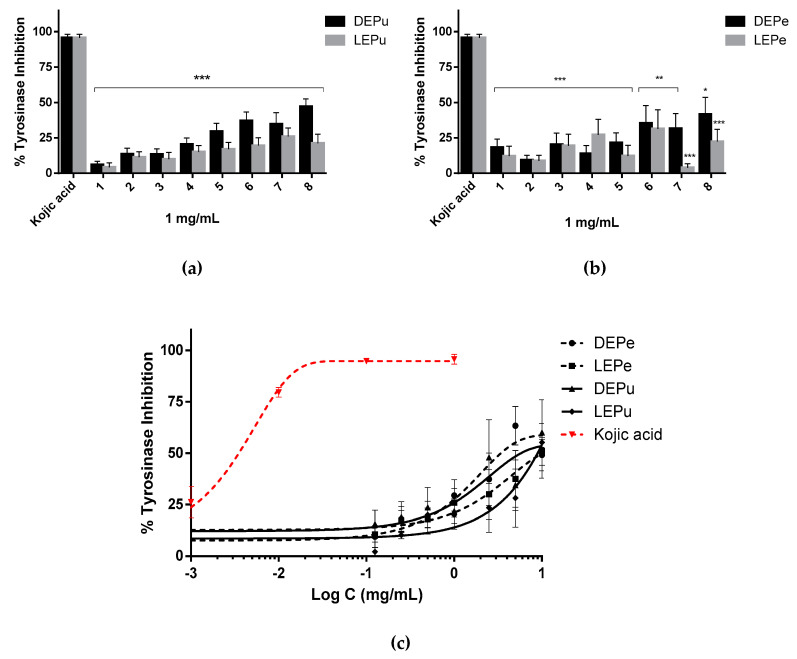
TYR inhibition: (**a**) inhibition performed by extracts obtained from pulp (*n* = 3); (**b**) inhibition performed by extracts obtained from peel (*n* = 3); (**c**) inhibition performed by sample 5 for DEPe and DEPu, sample 6 for LEPe and LEPu, and kojic acid in a concentration dependent manner (*n* = 3). One-way analysis of variance (ANOVA) followed by Dunnet’s test for multiple comparisons: * *p* < 0.05; ** *p* < 0.01; *** *p* < 0.001 against kojic acid. DEPe: dried peel methanolic extract; LEPe: lyophilized peel methanolic extract; DEPu: dried pulp methanolic extract; LEPu: lyophilized pulp methanolic extract.

As can be noted from the following figures (Figure 5a,b), the extracts were not able to inhibit significantly TYR at concentration of 1 mg/mL (inhibition less than 50%), and increasing the concentration to 10 mg/mL, the percentage remains almost the same (between 49% and 60%). IC_50_ values were calculated by nonlinear regression (Table 3).

### 2.3. Correlation Analysis between Phytochemical Composition and Bioactivity

In order to establish a relationship between phytochemical content and the enzymatic inhibitory capacity of the extracts, correlation analyses were performed for each type of extract. Results showed a strong negative correlation between the content of triterpenes and MAO-A inhibition (Pearson *r* Value = −0.8241) in LEPe, without a good correlation between polyphenols and the MAO-A bioassay (Pearson *r* Value = −0.2331). A strong positive correlation was found in the DEPu sample between the content of phenolic compounds and tyrosinase inhibition (Pearson *r* Value = 0.7924). Additionally, a very strong positive correlation in the analysis was found for the triterpene content versus lipase inhibition (Pearson *r* Value = 0.9568) in the same sample (Table 4).

## 3. Discussion

Two methods were used in this study for the dehydration of the fresh fruit: drying at 45 ± 5 °C and freeze-lyophilization. HPLC-DAD analysis revealed a higher content of phytochemicals in the LEPe and LEPu than in the DEPe and DEPu that can be due to the neutralization of the degradative enzymes by liquid nitrogen (−195.8 °C) used during the crushing of the fresh samples [2]. The peel extracts showed the highest content in polyphenols than the pulp ones because of the accumulation of these compounds in the peel according to the ecological role of this part such as protection against ultraviolet radiations, attraction for fruit dispersion and defense against pathogens [20,21]. Indeed, it is also known that triterpenes are concentrated on the surface of fruit peel [22]. This confirms the high concentrations detected in our samples. The variability of the total polyphenols and triterpenes content in extracts obtained from dried and freeze-lyophilized materials showed that the dehydration method used can influence the phytochemical content of a sample and is thus an important parameter to consider during the preparation of samples.

An increasing interest in the utilization of natural products as candidates for drug discovery, coadjuvant or alternative to drugs (food supplements, nutraceuticals) in the treatment of different pathologies has been demonstrated in the last years [23]. Thus, the present study was conducted in order to evaluate the nutraceutical or pharmaceutical potential of an old Italian apple variety. In particular, it was assayed the possible inhibitory effect of the apple extracts towards the enzymes α-glucosidase (α-GLU), lipase, monoamine oxidase A (MAO-A), tyrosinase (TYR) and acetylcholinesterase (AChE). These enzymes are related to pathologies such as diabetes, obesity, neurodegenerative disorders and melanogenesis. The phytochemical composition, in terms of concentration of phenolics and triterpenes [24], is strictly related to the inhibition of these enzymes. 

In our investigation, the extracts from dried peels inhibited better α-GLU than the ones from freeze-lyophilized peels, especially the samples 5 and 6 that showed a greater activity than the reference compound although without significant differences. This may be the result of the formation of some bioactive compounds during apple drying as reported by Birtic et al. [25].

As known α-GLU and lipase are digestive tract enzymes, involved in the metabolism of carbohydrates and fats, respectively. The inhibition of α-GLU can thus have an impact on diabetes treatment due to the reduction of intestinal absorption and decrease of post-lunch insulin values, maintaining the glycemic variations under control [26]. It is reported that the preventive effects of polyphenols against diseases such as diabetes and obesity may be the results of the modulation of receptors and enzymes such as α-GLU and lipase [10]. Some flavan-3-ols (e.g., catechin) showed an inhibitory effect against the enzymes α-GLU and lipase [27]. However, according to our results, we did not find a positive correlation for polyphenols and α-GLU inhibition.

It is reported that procyanidins inhibit the gastrointestinal lipase, thus decreasing the plasma triglycerides [28]. Triterpenes such as ursolic acid are known to significantly inhibit the pancreatic lipase [29]. In fact, a very strong positive correlation was found in this study between triterpenes and lipase inhibition in the DEPu samples, which reveals that these compounds are responsible for this activity at least in that extract. Quercetin and other flavonoids contributed significantly to the inhibition of the MAO-A, as well their antioxidant activity is related to the central protective action [30]. The polyphenols contained in the apple are able to cross the brain-blood barrier and for this reason showed antidepressant activity [31]. It has previously been reported that the flavonol quercetin and some polyphenol-rich extracts are able to inhibit the enzymes anticholinesterase (AChE) and butyrylcholinesterase (BChE), demonstrating thereby neuroprotective effect against pathologies such as Alzheimer’s disease [9]. It is noteworthy to mention that in spite of the use of the pulp for food and nutritive purposes, the peel contains a higher proportion of phytochemicals exerting better bioactive potential. There are no significant differences in triterpenes composition between the crude methanolic extracts prepared from dry material and the ones prepared from the freeze-lyophilized material, although the latter showed higher concentrations of polyphenols. Thus, the complex phytochemical composition and the synergism of each phytochemical in the extracts can be the result of the activity seen in the study [32]. The high phytochemical content of the peel extracts might justify their effectiveness with respect to pulp samples and their use for pharmaceutical applications.

The maximum activities achieved by the different extracts were at high concentrations. Although these doses are not physiological, these are in vitro studies, where the ability of the extracts to interact with the different enzymes is confirmed. In order to corroborate this action at a physiological level, subsequent studies should be considered.

## 4. Materials and Methods 

### 4.1. Sampling and Preparation of Apple Extracts

For this study, 8 apples (8 samples) (Table 5) of the cultivar Mela Rosa dei Monti Sibillini cultivated at an altitude between 250 and 500 m a.s.l. and harvested in October–November 2018 were collected from different farmers of the Montedinove, Montottone and Monterinaldo municipalities in the Marche region, Central Italy. The peel of the fruits (at least 5 fruits for each population of apples) was separated from the pulp. A portion (pulp and peel) was dried at 45 ± 5 °C for at least 18 h using a Biosec De Luxe B12 dryer (Albrigi Luigi, Verona, Italy) while the other part was crushed in a mortar with liquid nitrogen and lyophilized until the material was well dehydrated (Buchi, Cornaredo, Italy). The dehydrated material was then powdered using 2 mm-size particles using an IKA-WERK MFC DCFH 48 (Staufen, Germany).

All the dehydrated materials (dried and freeze-lyophilized) were submitted to the same bioactive compounds extraction with methanol as solvent [33,34] using an ultrasound bath (Ultrasonic Falc, Trviglio, BG, Italy). After a sonication for 45 min at room temperature of the sample with methanol (ratio 1 g of dehydrated material with 5 mL of methanol) and filtration, the residue was recovered and submitted to a further sonication for 20 min (ratio 1 g of dehydrated material with 4 mL of methanol). The filtrates of both sonication were gathered and concentrated under vacuum at 35 °C for at least 1 h using rotavapor.

At the end of the process, 32 extracts were obtained: 8 extracts obtained from dry-peel (DEPe), 8 from lyophilized-peel (LEPe), 8 extracts from dry-pulp (DEPu), and 8 from lyophilized-pulp (LEPu).

### 4.2. HPLC-DAD Analysis

The analytical method was carried out following a previous study [5]. Briefly, Synergi Polar-RP C18 (4.6 mm × 250 mm, 4 µm) analytical column from Phenomenex (Chesire, UK) was used as stationary phase. The mobile phases were mixtures of (A) water (*v/v*) and (B) methanol both containing 0.1% formic acid with a flow rate of 1 mL/min, with gradient elution. Twenty compounds of different classes were assessed: flavan-3-ols (catechin, epicatechin, procyanidin A2, procyanidin B2, cyanidin-3-glucoside) flavonols (quercetin, rutin, kaempferol, quercetin-3-O-galactoside, kaempferol-3-glucoside), hydroxycinnamic acids (chlorogenic acid, neochlorogenic acid, caffeic acid, *p*-coumaric acid, *trans*-ferulic acid) hydroxybenzoic acids (gallic acid), dihydrochalcones (phloretin and phloridzin) and triterpene acids (ursolic and oleanolic acids). The HPLC-DAD-MS analysis was performed on Hewlett-Packard HP-1090 Series II (Palo Alto, CA, USA), equipped with a vacuum degasser, a binary pump, an autosampler and a model 1046A HP photodiode array detector (DAD) and a mass spectrometer detector Trap SL (Bruker, Billerica, MA, USA) equipped with an electrospray ionization (ESI) source.

### 4.3. Enzyme Inhibitory Activities

In all the enzyme inhibition assays, the concentration of 1 mg/mL was first used in order to compare the different samples extracts and the reference inhibitors. After this screening, one of the samples with the highest activity was selected to perform the dose response curves.

#### 4.3.1. Reagents and Chemicals

α-Glucosidase from *Saccharomyces cerevisiae*, *p*-nitrophenyl glucopyranoside (pNPG), lipase type II from porcine pancreas, *p*-nitrophenyl butyrate (pNPB), vanillic acid, 4-aminoantipyrine, horseradish peroxidase, tyramine, monoamine oxidase A (MAO-A), galantamine, acetylthiocholine iodide (ATCI), 5,5′-dithiobis(2-nitrobenzoic acid) (DTNB), Trizma base, acetylcholinesterase (AChE), l-DOPA and tyrosinase were supplied by Sigma-Aldrich (Madrid, Spain); clorgyline and α-kojic acid were acquired through Cymit quimica (Barcelona, Spain); MgCl_2_·6H_2_O, HCl, NaCl, potassium phosphate were from Panreac (Barcelona, Spain).

#### 4.3.2. α-Glucosidase Inhibition Assay

α-Glucosidase inhibition assay was performed following the previous method [35]. Each 96-well microplate contained: 50 μL apple extract or the reference at different concentration and 100 μL of α-GLU (1U/mL) in phosphate buffer (pH 6.9). After 10 min, 50 μL of PNPG (50 μL) were added and incubated for 20 min at 37 °C. The absorbance was read at 405 nm. The percentage inhibition of α-glucosidase was calculated using the following formula:

% inhibition = [(Abs_control_ − Abs_Sample_)/Abs_control_] × 100 (1)

Acarbose was used as a positive control and IC_50_ values were determined.

#### 4.3.3. Lipase Inhibition Assay

A lipase inhibition assay was performed according to a previous method [35]. The extract or orlistat (reference inhibitor) at different concentrations were mixed with 40 μL of lipase type II (2.5 mg/mL in Tris-Buffer, pH 7.0). After 10 min of incubation at room temperature, 20 μL of pNPB (10 mM) were added. A 96-Well microplate was incubated for 15 min at 37 °C and the absorbance was read a 405 nm. The percentage inhibition on lipase was calculated using Equation (1).

#### 4.3.4. *Monoamine oxidase (MAO-A)* Inhibition Assay

MAO-A inhibition assay was performed according to a previous protocol [12]. In each well the following reagents were added: 50 µL of apple extract or reference inhibitor (clorgyline) at different concentrations, 50 µL of chromogenic solution (0.8 mM vanillic acid, 417 mM 4-aminoantipyrine and 4 U/mL horseradish peroxidase in potassium phosphate buffer, pH = 7.6.), 50 µL of samples or reference inhibitor at different concentrations, 100 µL of tyramine (3 mM) and 50 µL of MAO-A (8 U/mL). Blanks and control wells were made. The absorbance was read at 490 nm every 5 min during 30 min. Clorgyline was used as reference inhibitor. The MAO-A inhibitory activity was calculated by the Equation (1).

#### 4.3.5. *Acetylcholinesterase (AChE)* Inhibition Assay

AChE inhibitory activity was calculated using Ellman’s method [36]. Each well contained a mixed solution of 25 µL ATCI (15 mM in Milipore water), 125 µL of DTNB (3 mM in buffer (Tris-HCl-NaCl-MgCl_2_·6H_2_O, pH = 8.0), 50 µL of buffer (50 mM Tris-HCl, pH = 8, 0.1% bovine serum), and 25 µL of apple extract or galantamine (reference). Finally, 25 µL of AChE (0.22 U/L) were added to start the reaction. Absorbance was read 13 times every 13 s at 405 nm. The inhibitory activities were expressed as the percentage inhibition and calculated with Equation (1). The IC_50_ was subsequently calculated.

#### 4.3.6. *Tyrosinase* Inhibition Assay

Tyrosinase inhibitory activity was measured using a described procedure [37]. Apple extract or the reference (α-kojic acid) (10 μL) was mixed with tyrosinase (40 μL), 80 µL of buffer (phosphate, pH = 6.8) and 40 µL of l-DOPA. The absorbance was read at 475 nm. The percentage inhibition of tyrosinase was calculated using Equation (1). IC_50_ was also calculated.

#### 4.3.7. Statistical Analysis

The experiments were carried out in three replicates on different days. Analysis was performed using GraphPad Prism v.6. The results are expressed as mean ± standard error (±SEM). The differences between the different extracts were analyzed using one-way analysis of variance (ANOVA) followed by Dunnet’s test for multiple comparisons with a confidence interval of 95%. *p* values ≤ 0.05 were considered as significant differences. Pearson correlation analyses were performed in all samples (DEPu, LEPu, DEPe, LEPe) between polyphenol or triterpene content and bioactivity in terms of % of inhibition of the enzymes (α-GLU, lipase, MAO-A, AChE, tyrosinase) at 1 mg mL^−1^. Correlation coefficients (*r*) and statistical significances were calculated using GraphPad Prism v.6.

## 5. Conclusions

This study investigated the phytochemical composition as well as the enzyme inhibitory properties of the extracts of an overlooked traditional apple, the Mela Rosa dei Monti Sibillini. Its polar extracts, especially those obtained from the peel have demonstrated a rich content of bioactive compounds such as flavan-3-ols, flavonols, dihydrocalchones, and triterpenes. The freeze-lyophilization dehydration method was more effective in maintaining the phenolic constituents than the drying method. Some extracts have demonstrated inhibitory properties against α-GLU, lipase and MAO-A. The extracts obtained from the peel dried material exert better bioactivities than those obtained from lyophilized material. These results thereby demonstrated that this variety is a potential source of bioactive compounds for the production of pharmaceuticals or nutraceuticals to be used for the prevention and co-treatment of pathologies such as diabetes, obesity, Alzheimer’s disease, depression, and hypermelanosis.

## Figures and Tables

**Table 1:** Concentrations of phytochemicals, expressed in mg/kg, in the extracts obtained from the dried and lyophilized peel of Mela Rosa dei Monti Sibillini.

	Dried Samples								Lyophilised Samples							
	1	2	3	4	5	6	7	8	1	2	3	4	5	6	7	8
**Hydroxybenzoic Acids**																
Gallic Acid	8.8	6.8	25.5	7.9	6.7	8.4	3.9	10.2	6.5	3.9	4.1	4.1	8.2	0.0	0.0	0.0
**Flavan-3-ols**																
Catechin	333.5	136.1	190.4	223.1	309.7	301.2	221.2	364.2	470.0	235.9	260.8	260.4	552.0	426.2	320.4	403.0
Epicatechin	2042.9	938.1	1280.1	1257.0	1772.0	2083.5	1630.1	2049.9	2735.5	1740.2	3059.2	3053.9	3999.0	3976.4	3007.5	2869.5
Procyanidin B2	2041.4	855.0	858.5	874.0	1324.2	1177.9	1204.6	1201.6	2089.8	1655.9	2266.4	2262.5	2732.7	2421.4	2386.6	1995.1
Procyanidin A2	252.4	230.1	209.0	280.9	224.6	174.0	174.9	222.1	301.5	272.7	715.7	714.4	565.0	503.8	562.8	267.5
**Anthocyanins**																
Cyanidin 3-glucoside	27.7	7.1	16.9	19.7	19.3	9.8	7.7	9.8	50.8	51.9	103.5	103.3	79.4	123.7	43.9	39.2
**Flavonols**																
Rutin	306.2	572.2	509.9	656.8	342.0	687.2	553.2	545.1	0.0	882.0	1034.5	1032.7	972.9	1384.9	1422.4	917.6
Quercetin-3-O-galactoside	101.1	235.6	359.3	496.8	360.4	444.0	220.2	363.3	11.5	429.8	751.3	749.9	1313.8	1158.8	922.3	650.6
Kaempferol-3-glucoside	258.2	293.0	408.9	253.6	340.8	197.2	126.3	272.1	358.6	486.9	811.8	810.3	1015.7	472.4	336.6	296.7
Quercetin	6.2	5.2	16.6	6.5	6.7	3.8	8.0	14.6	21.3	9.1	14.4	14.3	8.5	4.3	14.4	20.9
Kaempferol	2.8	0.0	0.0	0.0	0.0	0.0	0.0	2.6	6.9	0.0	0.0	0.0	0.0	7.9	0.0	6.0
**Hydroxycinnamic Acids**																
Neochlorogenic Acid	6.0	0.0	0.0	7.2	4.4	4.5	4.2	4.3	12.0	0.0	0.0	0.0	9.8	0.0	4.2	5.8
Chlorogenic Acid	120.9	1551.7	84.0	90.2	143.6	163.7	137.1	196.0	240.9	1726.3	97.8	97.7	154.9	167.2	109.8	156.6
Caffeic Acid	21.0	0.0	13.6	18.7	11.7	13.0	18.8	18.6	26.2	0.0	0.0	0.0	14.9	5.5	17.4	19.9
*p*-Coumaric Acid	4.8	7.6	0.0	4.7	3.4	2.2	0.0	0.0	2.3	0.0	0.0	0.0	6.2	3.7	2.1	0.0
*trans*-Ferulic Acid	3.7	2.2	9.6	6.0	7.3	2.9	6.1	8.0	13.8	5.1	39.7	39.7	15.3	13.0	10.7	5.9
**Dihydrochalcones**																
Phloridzin	1315.0	507.3	527.1	387.7	600.5	872.8	398.5	562.9	1809.5	892.0	1628.3	1625.5	1228.1	2012.1	730.2	773.8
Phloretin	16.1	0.0	10.9	3.2	3.2	10.4	11.7	0.0	3.7	0.0	27.4	27.4	3.3	6.1	3.1	0.0
**Total Polyphenols**	6868.8	5347.9	4520.3	4593.9	5480.3	6156.5	4726.6	5845.2	8160.7	8391.5	10,814.9	10,796.1	12,679.8	12,687.5	9894.5	8427.8
**Triterpenes**																
Oleanolic Acid	6301.5	3996.2	598.5	3361.9	2340.1	3132.9	6527.3	3543.2	811.6	10,835.5	321.1	320.5	1398.0	6194.2	4658.1	4278.2
Ursolic Acid	15,088.8	10,719.5	1361.7	5383.3	2121.7	7188.4	9006.2	7307.5	1911.8	7667.8	584.4	583.4	3366.3	12,541.8	8722.0	9069.4
**Total Triterpenes**	21,390.4	14,715.8	1960.2	8745.2	4461.8	10,321.4	15,533.5	10,850.6	2723.5	18503.3	905.5	903.9	4764.3	18,736.0	13,380.1	13,347.6

**Table 2:** Concentrations of phytochemicals, expressed in mg/kg, of the extracts obtained from the dried and lyophilized pulp samples of Mela Rosa dei Monti Sibillini.

	Dried Samples								Lyophilised Samples							
	1	2	3	4	5	6	7	8	1	2	3	4	5	6	7	8
**Hydroxybenzoic Acids**																
Gallic Acid	4.8	5.7	3.9	3.8	5.6	6.4	4.1	11.8	2.4	2.2	0.0	2.7	2.0	2.0	5.7	4.9
**Flavan-3-ols**																
Catechin	183.5	131.7	261.8	144.9	285.3	336.6	271.3	366.1	214.5	164.8	176.2	184.9	340.1	236.7	195.1	416.9
Epicatechin	586.9	616.5	791.7	676.1	982.9	1208.7	820.5	1410.9	833.7	494.5	881.1	814.3	1292.2	738.7	769.3	1725.2
Procyanidin B2	338.9	325.6	455.4	429.2	827.5	714.0	472.0	1015.6	431.0	387.0	538.6	471.5	784.3	503.7	535.1	1262.2
Procyanidin A2	30.0	27.2	38.0	41.0	68.9	65.2	39.3	88.0	66.5	32.6	49.3	53.0	97.6	33.8	55.9	106.7
**Anthocyanins**																
Cyanidin 3-glucoside	0.0	0.0	0.0	0.0	0.0	0.0	0.0	0.0	0.0	0.0	0.0	0.0	0.0	0.0	0.0	0.0
**Flavonols**																
Rutin	88.9	63.5	82.9	65.3	99.5	125.8	86.0	123.9	108.7	84.5	109.7	71.4	117.4	82.7	61.7	124.7
Quercetin-3-O-galactoside	7.1	3.8	8.7	8.1	14.8	8.0	9.0	13.1	9.9	4.1	8.8	9.1	14.8	6.8	10.7	20.4
Kaempferol-3-glucoside	24.6	17.5	36.9	24.7	48.5	30.3	38.3	36.4	37.0	19.3	49.2	27.4	73.1	32.2	22.7	55.4
Quercetin	0.0	0.0	0.0	0.0	0.0	0.0	0.0	0.0	0.0	0.0	0.0	0.0	0.0	0.0	0.0	0.0
Kaempferol	0.0	0.0	0.0	0.0	0.0	0.0	0.0	0.0	0.0	0.0	0.0	0.0	0.0	0.0	0.0	0.0
**Hydroxycinnamic Acids**																
Neochlorogenic Acid	2.7	2.4	3.0	1.9	4.3	3.3	3.1	5.8	3.1	3.5	2.3	4.0	4.3	4.3	4.7	7.1
Chlorogenic Acid	311.9	1438.7	306.5	136.0	260.8	492.0	317.7	529.6	384.2	1946.8	244.6	228.2	273.6	387.5	167.9	338.8
Caffeic Acid	14.0	0.0	9.8	16.1	15.2	21.5	10.1	0.0	14.6	0.0	14.6	14.3	26.5	15.9	21.0	16.5
*p*-Coumaric Acid	1.0	59	0.0	1.0	1.1	1.1	0.0	1.0	0.0	6.1	0.0	1.1	1.1	1.0	1.0	0.8
*trans*-Ferulic Acid	1.1	1.2	1.1	1.5	1.1	1.6	1.1	1.8	2.0	2.5	4.5	1.6	1.8	1.6	1.2	1.8
**Dihydrochalcones**																
Phloridzin	72.1	73.1	61.3	49.6	64.5	101.0	63.5	64.5	136.9	135.5	98.5	68.8	122.4	97.3	58.7	103.7
Phloretin	0.0	0.0	0.0	0.0	0.0	0.0	0.0	0.0	0.0	0.0	0.0	0.0	0.0	0.0	0.0	0.0
**TOTAL Polyphenols**	1667.6	2712.9	2060.9	1599.0	2679.7	3115.4	2135.9	3668.5	2244.3	3283.4	2177.4	1952.3	3151.2	2144.0	1910.7	4185.0
***Triterpenes***																
Oleanolic Acid	47.7	123.6	144.6	71.9	85.3	95.0	149.9	32.7	39.7	107.7	107.2	142.5	75.8	56.6	178.9	19.7
Ursolic Acid	40.6	29.8	77.4	29.4	137.7	36.2	80.2	48.3	35.5	17.3	47.2	82.9	90.7	58.0	23.3	37.5
**Total Triterpenes**	88.3	153.4	222.0	101.2	223.0	131.1	230.0	81.0	75.2	125.0	154.4	225.3	166.5	114.6	202.2	57.2

**Table 3:** IC_50_ values (μg/mL) of different extracts in different tested bioassays.

IC_50_ Value in µg/mL (Mean ± SEM)					
Bioassay	DEPe	LEPe	DEPu	LEPu	Reference Inhibitor
**α-glucosidase**	1417 ± 195	1056 ± 168	-	-	379 ± 74 (Acarbose)
	Ns		-		**/*/-/-
**Lipase**	3690 ± 2503	3375 ± 3690	-	4783 ± 237	0.90 ± 0.01 (Orlistat)
	Ns		-		ns/ns/-/ns
**MAO-A**	361 ± 11	291 ± 26	797 ± 4	650 ± 14	0.15 ± 0.02 (Clorgyline)
	^#^		^&&&^		****/****/****/****
**AChE**	1887 ± 211	2261 ± 574	3963 ± 431	-	0.56 ± 0.01 (Galantamine)
	Ns		-		*/*/***/-
**TYR**	-	3419 ± 145	8953 ± 124	3690 ± 1154	3.81 ± 0.22 (Kojic acid)
	-		^&&&^		-/*/****/**

MAO-A: monoamine oxidase A; AChE: acetylcholinesterase; TYR: tyrosinase; DEPe: dried peel methanolic extract; LEPe: lyophilized peel methanolic extract; DEPu: dried pulp methanolic extract; LEPu: lyophilized pulp methanolic extract. Data were analysed using one-way ANOVA multiple comparisons; ns: not significant; ^#^ *p* < 0.05 between DEPe and LEPe; ^&&&^ *p* < 0.001 between DEPu and LEPu; * *p* < 0.05 or ** *p* < 0.01 or *** *p* < 0.001 or **** *p* < 0.0001 between DEPe, LEPe, DEPu, or LEPu and the reference inhibitor.

**Table 4:** Pearson *r* values and statistical significance for correlation analyses between polyphenol or triterpene contents of the different samples (DEPe, LEPe, DEPu, LEPu) and bioassays.

Bioassay	DEPe		LEPe		DEPu		LEPu	
	*Phenol.*	*Triterp.*	*Phenol.*	*Triterp.*	*Phenol.*	*Triterp.*	*Phenol.*	*Triterp.*
**α-GLU**	0.1142	−0.4741	0.2428	−0.09464	−0.2745	−0.0469	0.3865	−0.2265
	ns	ns	ns	ns	ns	ns	ns	ns
**Lipase**	−0.07635	−0.3406	0.1867	0.2813	−0.3416	0.9568	0.4261	−0.1890
	ns	ns	ns	ns	ns	***	ns	ns
**MAO-A**	0.1567	0.1037	−0.2331	−0.8241	0.5092	−0.2526	−0.4146	0.6902
	ns	ns	ns	*	ns	ns	ns	ns
**AChE**	−0.2922	0.2110	−0.1816	−0.5390	0.4546	−0.1949	−0.1910	−0.4477
	ns	ns	ns	ns	ns	ns	ns	ns
**TYR**	0.1816	−0.1827	0.3528	−0.3741	0.7924	−0.1270	0.1498	0.02118
	ns	ns	ns	ns	*	ns	ns	ns

ns: no significance; * *p* < 0.05; *** *p* < 0.001. DEPe: dried peel methanolic extract; LEPe: lyophilized peel methanolic extract; DEPu: dried pulp methanolic extract; LEPu: lyophilized pulp methanolic extract; *Phenol.*: polyphenol content; *Triterp.*: triterpene content.

**Table 5:** Main information on the apple samples of Mela Rosa dei Monti Sibillini.

Sample N.	Farmers	Variety	Altitude (m)	Graft	Municipality
**1**	Traini	classica	500	M111	Montedinove
**2**	Botticelli	classica	390	M111	Montottone
**3**	Orsolini	classica	480	M111	Montedinove
**4**	Galli Stefano	classica	250	M9 M26	Montedinove
**5**	Geminiani I	classica	450	M111	Montedinove
**6**	Geminiani II	classica	480	M111	Montedinove
**7**	Orsolini	pianella abruzzese	480	M111	Montottone
**8**	Acciarri	classica	350	M 26	Monterinaldo
